# T7-Synthesized Double-Stranded RNA Mimicking miR-71 Induces Termite RNAi and Increases Fungal Efficacy

**DOI:** 10.3390/biom15111517

**Published:** 2025-10-27

**Authors:** Chenchen Zhao, Hang Lu, Ruotian Cheng, Pengfei Zhao, Gaoling Zhang, Hongsong Chen, Qingbo Tang, Long Liu

**Affiliations:** 1 Henan International Laboratory for Green Pest Control, Henan Engineering Laboratory of Pest Biological Control, College of Plant Protection, Henan Agricultural University, Zhengzhou 450046, China; zhaochen@henau.edu.cn (C.Z.);; 2 Guangxi Key Laboratory of Biology for Crop Diseases and Insect Pests, Institute of Plant Protection, Guangxi Academy of Agricultural Sciences, Nanning 530007, China; 3 Henan Academy of Architectural Heritage Conservation and Research, Zhengzhou 450011, China

**Keywords:** *Metarhizium anisopliae*, miR-71, transcriptomics, gene silencing, synergistic biocontrol

## Abstract

miR-71 has been determined to enhance the efficacy of biological control agents against termites. However, it is not clear how miR-71 functions in enhancing the termite control. In this study, we tested the effects of termite miR-71 on the transcriptional and translational profiles of termites via the commercial product miR-71 agomir, and meanwhile developed a cost-effective method using T7 RNA polymerase to synthesize a miR-71 mimic, comparing the effects of the T7-synthesized miR-71 mimic versus the commercial miR-71 agomir on the gene expressions and infection mortality of termites. Comparative bioassays demonstrated that both miR-71 mimic and agomir significantly increased fungus-induced termite mortality with equivalent bioactivity. Mechanistically, transcriptomic and proteomic analyses revealed that commercial miR-71 agomir modulated the expression of defense-related genes, such as *hexamerin-1*, *neuroligin-4*, and *probable chitinase*-10. Meanwhile, RT-qPCR confirmed that T7-synthesized miR-71 mimic induced similar expression changes in the same target genes. Additionally, the dsRNA-mediated silencing of *hexamerin-1*, *neuroligin-4*, and *probable chitinase*-10 made termites more vulnerable to the fungus, respectively. Our study establishes in vitro-transcribed miRNA mimics as potent and cost-effective tools for studying ‘miRNA–mRNA’ interaction, and meanwhile lays the foundation for the microbe-mediated expression of small-RNA mimics in enhancing termite biocontrol.

## 1. Introduction

Insect pests pose a persistent and significant threat to global agriculture, forestry, and public health [1]. Species such as termites cause extensive structural damage by feeding on wood and cellulose-rich materials, leading to substantial economic losses annually [2]. Beyond their direct impact, many insect pests act as vectors for plant pathogens, contributing to crop yield reductions and further compromising food security worldwide [3].

For decades, chemical insecticides have been the primary tool for pest control. While often effective, their widespread and indiscriminate use has precipitated a cascade of adverse consequences [4], including the evolution of pesticide resistance, pest resurgence, the disruption of ecological balances, and the contamination of soil and water resources [5]. These impacts extend to non-target organisms, harming beneficial insects like pollinators and natural predators, thereby underscoring the urgent need for more sustainable and environmentally benign pest management strategies.

One promising alternative is the use of entomopathogenic fungi as biological control agents [6]. Fungal pathogens such as *Metarhizium anisopliae* and *Beauveria bassiana* naturally infect a wide range of insect hosts, penetrating the cuticle and proliferating within the hemocoel, which ultimately leads to host death [7]. These fungi offer key advantages, including species specificity, environmental safety, and compatibility with integrated pest management (IPM) strategies [8]. However, their effectiveness can be constrained by environmental factors and host resistance, necessitating innovative approaches to enhance their virulence [9].

RNA interference (RNAi) has emerged as a transformative technology for potentiating the efficacy of fungal biocontrol [10]. As a natural posttranscriptional gene silencing mechanism triggered by double-stranded RNA (dsRNA) or small RNAs, RNAi can be harnessed to suppress critical insect genes, thereby increasing susceptibility to pathogens [11]. The delivery of dsRNA, either by injection or oral feeding, has successfully silenced genes involved in immunity, detoxification, and development, leading to a synergistic increase in mortality when combined with fungal infection [12,13]. For instance, silencing genes essential for chitin synthesis or antioxidant defense in termites has been shown to significantly accelerate mortality induced by *M. anisopliae* [14,15].

Building on this principle, miRNA-mediated RNAi offers an even more sophisticated approach. MicroRNAs (miRNAs) are small (20–24 nucleotide) non-coding RNAs that function as master regulators of gene expression, often targeting entire networks of genes involved in critical physiological processes like immunity, metabolism, and stress response [16]. Their ability to modulate complex biological pathways positions them as ideal targets for developing novel strategies to weaken host defenses [17,18]. Synthetic small RNAs designed to mimic endogenous miRNAs, such as miR-71, can thus act as powerful tools to manipulate insect physiology and amplify the effects of biological control agents [19].

The primary tools for studying miRNA function in vivo are commercially synthesized mimics and agomirs—chemically modified small RNAs designed for enhanced stability and cellular uptake [20]. While highly effective for functional studies, the high synthesis cost of these molecules severely restricts their practical application in large-scale RNAi experiments and even pest management programs [21]. This economic barrier highlights a critical need for simple, low-cost, and scalable methods to produce small functional RNAs for insect researchers. Large numbers of studies use biological promoters, such as T7, T3, SP6, and U6 promoters, to generate dsRNA, siRNA, or shRNA in vivo or in vitro, which is cost-efficient and contributes to the high-throughput screening of novel functional or target genes [22]. However, whether these promoter-generated exogenous siRNA can mimic endogenous miRNA in insect study and biocontrol has yet to be understood completely [23].

Our preliminary studies suggest that T7 RNA polymerase-synthesized small double-stranded RNAs based on the sequence of miR-71 can significantly suppress antimicrobial activity in termites and enhance the efficacy of entomopathogenic fungi [15], indicating that T7-synthesized miR-71 mimic has the bioactivity in termites. However, whether this siRNA functions in the same manner as the endogenous miR-71 remains to be fully elucidated. To validate the biological activity of this cost-effective approach, we designed a comprehensive, multi-tiered investigation. First, we directly compared the synergistic effects of our T7-synthesized miR-71 mimic with a commercial miR-71 agomir on termite mortality following fungal challenge. Next, we employed transcriptomics and proteomics to map the global gene expression changes induced by the miR-71 agomir, screening miR-71-targeted genes. Meanwhile, we used RT-qPCR to confirm that our T7-synthesized mimic induced similar expression changes in these target genes. Finally, we performed functional gene-silencing assays to confirm that the identified target genes are directly responsible for increased fungal susceptibility. This comprehensive approach provides robust evidence for the functional equivalence of T7-synthesized mimics and lays the foundation for the expression and application of endogenous small-RNA mimics in fungal biocontrol.

## 2. Materials and Methods

### 2.1. Insect Rearing

All termites used in this study were worker castes of *Reticulitermes aculabialis*, collected from a natural population in Xinyang City, Henan Province, China. Following collection, termites were maintained under controlled laboratory conditions in group colonies. They were housed in ventilated plastic containers (40 × 20 × 20 cm) containing moistened pine wood blocks as both the shelter and food source. The rearing environment was kept at 25 ± 1 °C with 80% relative humidity under continuous darkness to simulate their natural subterranean habitat and ensure colony cohesion as previous reported.

### 2.2. Entomopathogen Cultivation

The entomopathogenic fungus *M. anisopliae* (strain IBCCM321.93) was cultured on potato dextrose agar (PDA) plates and incubated at 25 ± 1 °C with 80% relative humidity under continuous darkness for a period of 14 days to allow conidial maturation. Conidia were harvested by gently scraping the surface of the cultures and suspended in sterile 1% (*v*/*v*) Tween 80 solution. The resulting conidial suspension was stored at 4 °C for no longer than 3–4 weeks prior to use. Germination rates were routinely assessed before each experimental application, and only suspensions exhibiting greater than 90% germination were employed for termite inoculation assays.

### 2.3. Synthesis of miRNA Mimics and dsRNA

MiR-71 agomir were directly generated by RIBOBIO company (China, Guangzhou). The T7-synthesized miR-71 mimic was designed based on the termite miR-71 sequence (tgaaag acatgg gtagtg aga) and synthesized as a short double-stranded RNA. The miR-71 templates used for in vitro transcription were prepared via touchdown PCR using two sets of oligonucleotide pairs containing T7 promoters. The first template was generated from oligonucleotides A1 (5′-GATCAC TAATAC GACTCA CTATAG GGtgaa agacat gggtag tgaga-3′) and A2 (5′-tctcac taccca tgtctt tcaCCC TATAGT GAGTCG TATTAG TGATC-3′); the second template was prepared from B1 (5′-GATCAC TAATAC GACTCA CTATAG GGtctc actacc catgtc tttca-3′) and B2 (5′-tgaaag acatgg gtagtg agaCCC TATAGT GAGTCG TATTAG TGATC-3′). A control miRNA mimic based on a 19 nt fragment from the *GFP* gene was similarly designed according to Liu et al. (2023a, b) [15,24]. All mimics were transcribed using the T7 RNAi Transcription Kit and extracted with phenol–chloroform–isoamyl alcohol (50:49:1), followed by concentration and purity assessment via gel electrophoresis and NanoDrop. The resulting miRNA mimics were stored at −80 °C prior to use.

For dsRNA generation, a 483 bp fragment of *hexamerin-1* (Forward: TCAAGA CCGCAT ACAACC CC; Reverse: CCGTCA CTCCTT CGAACC AA), a 532 bp fragment of *Neuroligin-4* (Forward: CATGGC CTTAGC CCTTGA GT; Reverse: ACCATA AAGTGC AGGTCC AGT), a 262 bp fragment of *Probable chitinase 10* (Forward: GCGACT GCCATT GGTTCT TC; Reverse: CCATCA GGGCAC TTAGTG GT), and a 467 bp fragment of *GFP* (used as control; Forward: CTTGAA GTTGAC CTTGAT GCC; Reverse: TGGTCC CAATTC TCGTGG AAC) were amplified using primers containing the T7 promoter sequence at their 5′ ends (5′-GATCAC TAATAC GACTCA CTATAG GG-3′). PCR products were extracted using phenol–chloroform–isoamyl alcohol (25:24:1). dsRNA was synthesized with the T7 RNAi Transcription Kit and purified using phenol–chloroform–isoamyl alcohol (50:49:1). The purity and concentration of dsRNA were confirmed by agarose gel electrophoresis and NanoDrop 2000 spectrophotometry ((Thermo Scientific, Wilmington, DE, USA), and dsRNAs were stored at −80 °C until use.

### 2.4. RNA Injection and Fungal Contamination

According to the experiment method provided by Liu et al. (2020) [25], termites were injected with 1 µg of either miRNA or dsRNA and reared for 48 h in a plastic Petri dish (d = 35 mm) containing a moistened filter paper before fungal contamination. After two days of recovery, the injected termites were contaminated with conidia suspension (~10^7^ conidia/mL) for RNA-Seq (*n* = three replicates), RNAi efficiency (miRNA: *n* = three replicates; dsRNA: *n* = four replicates), and mortality assays (*n* = 30 individuals per treatment), respectively.

### 2.5. RNA Extraction, Library Preparation, and RNA-Seq

Total RNA from termite whole bodies (*n* = 10 termites per replicate, three replicates in total) was extracted using TRIzol™ Reagent (Invitrogen) according to the manufacturer’s instructions. RNA integrity was confirmed with an Agilent 2100 Bioanalyzer (RIN > 7.0). cDNA libraries were prepared using the NEBNext^®^ Ultra™ RNA Library Prep Kit (NEB, New England BioLabs, Inc., Ipswich, MA, USA) and sequenced on the Illumina NovaSeq 6000 platform (paired-end, 150 bp). For de novo assembly, clean reads were filtered and assembled using Trinity v2.14.0. Transcript quantification was performed with RSEM v1.3.3, and differential expression analysis was conducted with DESeq2 (|log_2_ fold change| ≥ 1, adjusted *p* < 0.05). Functional annotation and enrichment analyses were performed using BLASTx (https://blast.ncbi.nlm.nih.gov/, accessed on 16 June 2025, NR, Swiss-Prot, KEGG, GO).

### 2.6. RT-qPCR Analysis

Gene expression analysis was conducted using quantitative real-time PCR (RT-qPCR). For each replicate and treatment group, total RNA was extracted from pooled samples of three termites using the Direct-zol™ RNA MiniPrep Kit (Zymo Research, Irvine, CA, USA), following the manufacturer’s instructions. The concentration and purity of the RNA were assessed using a NanoDrop 2000 spectrophotometer (Thermo Fisher Scientific, Waltham™, MA, USA). First-strand cDNA synthesis was performed with the PrimeScript™ RT Reagent Kit with gDNA Eraser (Perfect Real Time) (Takara Bio Inc., Otsu, Japan), ensuring the removal of genomic DNA contamination. RT-qPCR reactions were carried out on a QuantStudio™ 3 Real-Time PCR System (Thermo Fisher Scientific, Waltham™, MA) using the ChamQ Universal SYBR qPCR Master Mix (Vazyme Biotech Co., Ltd., China). Primer sequences used for target and reference genes are provided in Table S1.

### 2.7. TMT-Based Quantitative Proteomics

For protein extraction, termite samples (*n* = 50) were ground in liquid nitrogen and lysed in 8 M urea buffer containing protease inhibitors. Protein concentration was determined via the BCA assay. Trypsin digestion was performed using the FASP method, and peptides were labeled with TMT 10-plex reagents (Thermo Fisher Scientific, Waltham™, MA, USA). Labeled peptides were fractionated via high-pH reversed-phase chromatography and analyzed on an Orbitrap Exploris 480 mass spectrometer coupled to an EASY-nLC 1200 system (Thermo Fisher Scientific, Waltham™, MA, USA). MS/MS data were processed with Proteome Discoverer 2.5 against the translated termite transcriptome database. Differentially abundant proteins (DAPs) were defined with |log_2_ fold change| ≥ 0.58 and adjusted *p* < 0.05, and annotated using GO and KEGG enrichment analyses.

### 2.8. Statistical Analysis

Survival curves were compared using the Kaplan–Meier method in GraphPad Prism 9. Different letters indicated significant differences at *p* < 0.05. Transcriptomic and proteomic enrichment analyses used Benjamini–Hochberg correction for multiple testing. RT-qPCR results were analyzed by paired *t* test. Data are presented as mean ± standard error (SE). Asterisks represented significant differences (*, *p* < 0.05; **, *p* < 0.01).

## 3. Results

### 3.1. Effects of T7-Synthesized miR-71 Mimic Vs. Mir-71 Agomir on Fungal Efficacy

**Figure 1:**
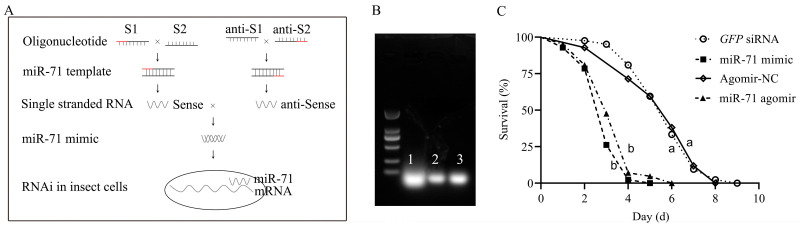
Schematic diagram of miR-71 mimic synthesis and its bioactivity assessment. (**A**) Experimental procedure for synthesizing miR-71 mimic using T7 transcription (Red: T7 promoter); (**B**) band of (1) miR-71 agomir and (2, 3) 71 mimic in agarose gel electrophoresis; (**C**) effects of miR-71 mimic and miR-71 agomir on the mortality of fungus-infected termites.

To establish a cost-effective alternative to commercial miRNA agomirs, we synthesized a functional miR-71 mimic using an in vitro T7 RNA polymerase transcription system (Figure 1A). Agarose gel electrophoresis showed the bands (Figure 1B). The process involved designing complementary oligonucleotide templates for miR-71, generating sense and antisense single-stranded RNAs, and annealing them to form a double-stranded miRNA mimic. The synthesized mimic was subsequently introduced into termites to assess its functional equivalence to a chemically modified miR-71 agomir in enhancing the pathogenicity of *M. anisopliae*.

Survival assays revealed that both the T7-synthesized miR-71 mimic and the miR-71 agomir significantly increased termite mortality following fungal infection compared with *GFP* siRNA and agimir-NC (negative control) (Figure 1C). Mortality in the miR-71 mimic and agomir groups reached >95% by day 4–5 post-infection, whereas control groups exhibited slower mortality kinetics, with complete death occurring by day 7–8. Kaplan–Meier analysis indicated significant differences in survival rates between RNAi-treated and control groups (*p* < 0.05). These results confirm that the T7-synthesized miR-71 mimic is capable of reproducing the insecticidal efficacy of commercial agomirs and, when combined with entomopathogenic fungi, substantially accelerates host mortality.

### 3.2. Effects of miR-71 Agomir on Termite Gene Expression Profiles

**Figure 2:**
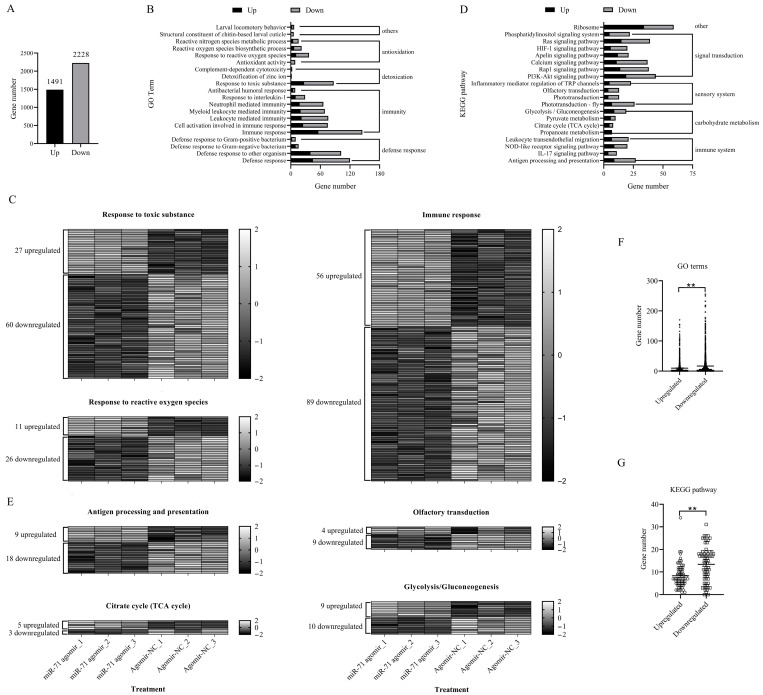
Effects of miR-71 agomir on termite gene expression profiles revealed by RNA-seq analysis. (**A**) Differentially expressed genes (DEGs); (**B**) Gene Ontology (GO) enrichment analysis of DEGs in response to miR-71 agomir, showing upregulated (black bars) and downregulated (gray bars) genes across major biological processes, including detoxification, immune response, and defense pathways; (**C**) heatmaps representing the expression profiles of genes involved in key biological responses, including toxic substance response, reactive oxygen species response, and immune response; (**D**) KEGG pathway enrichment analysis of DEGs, indicating significant enrichment in pathways related to signal transduction, immune function, and carbohydrate metabolism; (**E**) heatmaps of DEGs associated with specific KEGG pathways, including antigen processing and presentation, olfactory transduction, citrate cycle (TCA cycle), and glycolysis/gluconeogenesis; (**F**,**G**) GO and KEGG enrichment analysis of upregulated versus downregulated genes. Each column represents one replicate from miR-71 agomir or control treatments. **, *p* < 0.01.

To elucidate the molecular basis of miR-71-mediated enhancement of fungal efficacy, we performed transcriptome profiling of miR-71 agomir-treated termites compared with negative controls. The analysis revealed that a total of 3719 differentially expressed genes (DEGs) (1491 upregulated; 2228 downregulated) were determined in miR-71-treated termites compared to *GFP* siRNA-treated termites (Figure 2A and Table S2). GO enrichment revealed that DEGs were clustered into 1344 GO terms, including “response to toxic substance” (27 upregulated, 60 downregulated), “response to reactive oxygen species” (11 upregulated, 26 downregulated), and “immune response” (56 upregulated, 89 downregulated) (Figure 2B,C and Table S3). KEGG enrichment analysis further demonstrated that DEGs were mapped to 68 KEGG pathways, including “antigen processing and presentation” (9 upregulated, 18 downregulated), “olfactory transduction” (4 upregulated, 9 downregulated), “citrate cycle (TCA cycle)” (5 upregulated, 3 downregulated), and “glycolysis/gluconeogenesis” (9 upregulated, 10 downregulated) (Figure 2D,E and Table S4). Both GO terms and KEGG pathways exhibited a dominant trend of downregulation (*p* < 0.01), indicating the miR-71-mediated suppression of host immunity, detoxification, oxidative stress defense, and so on (Figure 2F,G).

### 3.3. RT-qPCR Verification for RNAi Mediated by T7-Synthesized miR-71 Mimic

**Figure 3:**
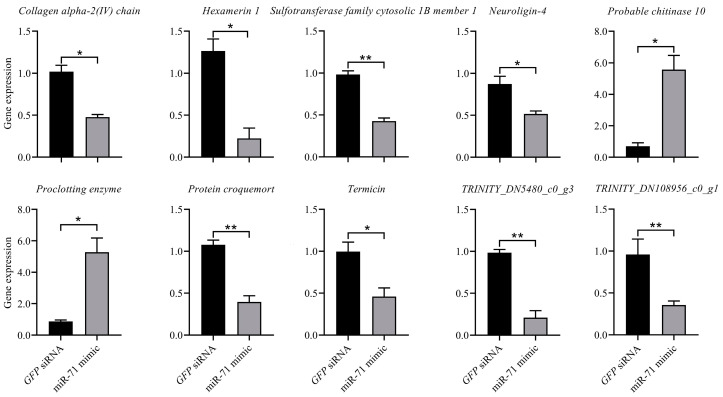
Effects of miR-71 mimic on defense-related gene expression validated by RT-qPCR. *, *p* < 0.05; **, *p* < 0.01.

To determine effects of T7-synthesized miR-71 mimic on miR-71-targeted gene expressions, based on our transcriptome findings, we selected 10 genes involved in signaling transduction and transmission (*Collagen alpha-2[IV] chain* and *Neuroligin-4*), cuticle resistance (*Probable chitinase 10*), defense response (*Hexamerin 1*, *Proclotting enzyme Protein croquemort*, and *Termicin*), and other functions for RT-qPCR analysis. Results showed that gene expressions, assessed by RT-qPCR verification, were consistent with results obtained by RNA-seq (Figure 3). Specifically, *collagen alpha-2(IV) chain*, *hexamerin 1*, *luciferin sulfotransferase*, *neuroligin-4*, *termicin*, and *protein croquemort* showed significant downregulation in the miR-71 mimic group compared with *GFP* siRNA controls (*p* < 0.05 or *p* < 0.01). In contrast, the miR-71 mimic can also markedly increase the expression of *probable chitinase 10* and *proclotting enzyme* compared to the controls (*p* < 0.05). The reduction in termicin expression is particularly notable given its established role as an antimicrobial peptide in termite defense.

### 3.4. Further Verification by Tandem Mass Tag (TMT)-Based Quantitative Proteomics

**Figure 4:**
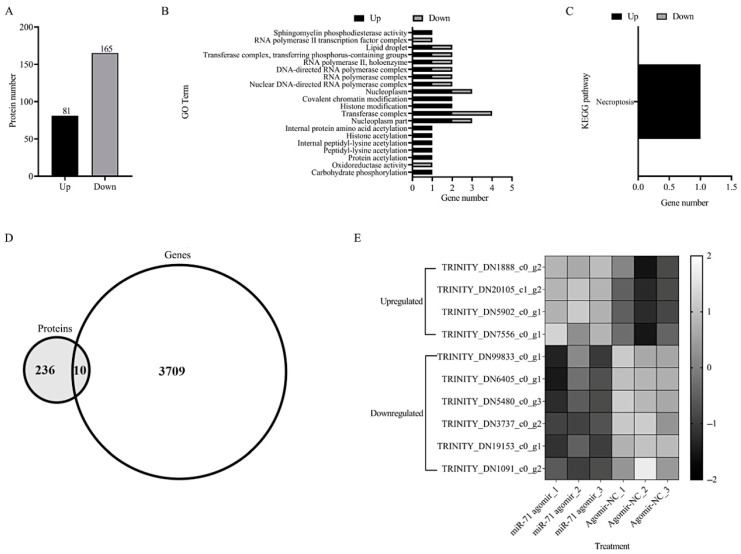
Effects of miR-71 agomir on termite protein expression profiles examined by tandem mass tag (TMT)-based quantitative proteomics. (**A**) Differentially expressed proteins affected by miR-71 agomir; (**B**) GO terms of differentially expressed proteins; (**C**) KEGG pathways of differentially expressed proteins; (**D**) Venn diagram of proteomic and transcriptomic datasets; (**E**) heatmap of protein expression for the overlapping genes in the Venn diagram.

To assess whether the transcriptional effects of miR-71 were reflected at the protein level, we conducted TMT-based quantitative proteomics on miR-71 agomir-treated termites. A total of 246 proteins were differentially expressed relative to controls, including 81 upregulated and 165 downregulated proteins (Figure 4A, Table S5). GO analysis indicated that these proteins were enriched in categories such as chromatin modification, RNA polymerase complexes, and histone modification (Figure 4B, Table S6), suggesting that miR-71 may modulate transcriptional machinery and epigenetic regulation. KEGG pathway enrichment identified necroptosis as a significantly affected pathway (Figure 4C, Table S7), suggesting that programmed cell death may be involved in the miR-71-mediated enhancement of fungal virulence.

The integration of transcriptomic and proteomic data revealed only 10 overlapping genes showing differential expression at both mRNA and protein levels (Figure 4D). This may be attributed to the fact that miR-71 may frequently regulate the expression of target genes at the posttranscriptional level through incomplete complementary pairing with mRNA. Heatmap visualization (Figure 4E) showed concordant expression trends—either up- or downregulation—for these genes across datasets (Tables S2 and S5). These findings suggest that miR-71 may exert coordinated regulatory effects at multiple molecular levels, although further studies are required to confirm direct causality and mechanistic pathways.

### 3.5. Effects of the Genes Targeted by miR-71 on Fungal Efficacy

**Figure 5:**
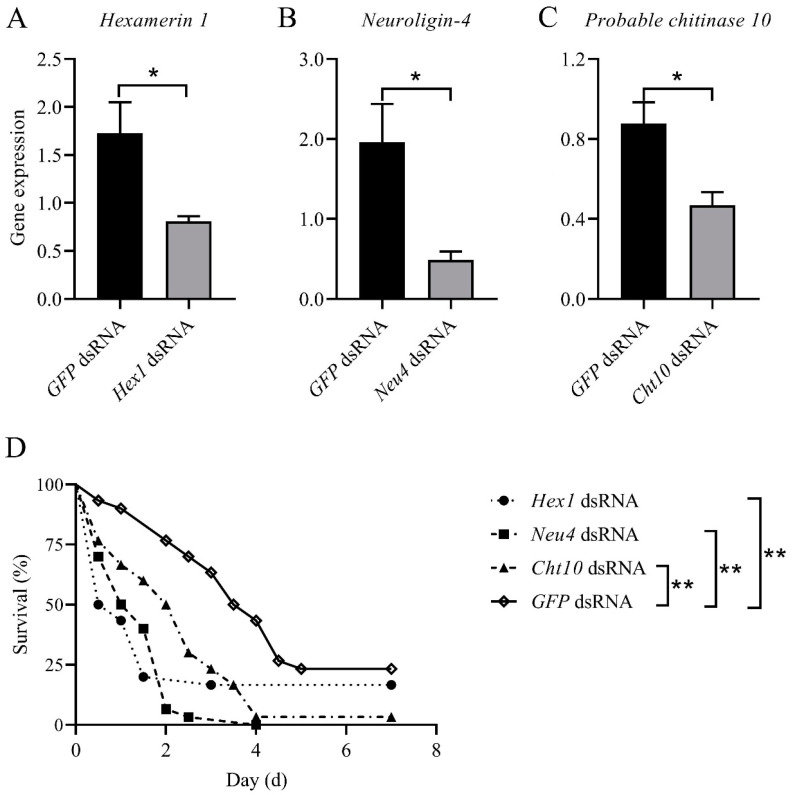
Effects of target genes on termite disease defense. Silencing efficiency of target genes *Hexamerin 1* (**A**), Neuroligin-4 (**B**), *Probable chitinase 10* (**C**); (**D**) Effects of target genes on termite mortality following infection. *, *p* < 0.05; **, *p* < 0.01.

To functionally confirm the relevance of miR-71 target genes to fungal pathogenicity, we performed gene-specific knockdowns of *hexamerin 1*, *neuroligin-4*, and *probable chitinase 10* via dsRNA-mediated RNAi (*p* < 0.05; Figure 5A–C). The silencing of *hexamerin 1* and *neuroligin-4*—both significantly downregulated by miR-71—led to pronounced reductions in termite survival following *M. anisopliae* infection. Knockdown of probable chitinase 10, which was upregulated by miR-71, also significantly reduced survival compared with *GFP* dsRNA controls (*p* < 0.01; Figure 5D). These functional assays demonstrate that miR-71’s enhancement of fungal virulence is mediated, at least in part, through modulation of specific target genes that regulate termite immunity, signaling transmission, and cuticle resistance. We also provide multi-tiered evidence that T7-expressed siRNAs can mimic endogenous miRNAs, which lays foundation for miRNA in the potentiating fungal biocontrol of termites.

## 4. Discussion

MicroRNAs (miRNAs) are endogenous single-stranded RNAs of 18~25 nt, which can target specific mRNA transcripts and prevent protein expression [26]. We found that an exogenous double-stranded RNA can mimic miR-71 and performed similar function in termite disease defense. Specifically, miR-71, delivered either as a chemically modified agomir or as an unmodified mimic synthesized via T7 RNA polymerase-mediated transcription, can significantly enhance the insecticidal efficacy of the entomopathogenic fungus *M. anisopliae* against termites. Through a multi-tiered approach consisting of survival bioassays, transcriptomic profiling, RT-qPCR validation, quantitative proteomics, and functional gene knockdowns, we determine that miR-71 modulates termite disease defense through multi-level interference with signaling transmission (e.g., *neuroligin-4*), cuticle resistance (e.g., *probable chitinase 10*), and immune response (e.g., *hexamerin 1* and *termicin*).

Recent studies have shown the inhibiting effects of miRNAs on insect disease resistance [27,28,29], suggesting the potential in RNAi-based pest control and biocontrol. Unlike miRNA agomirs, which require expensive chemical modifications to enhance stability and cellular uptake [30,31,32], T7-transcribed miRNA mimics are unmodified here, double-stranded RNAs [33,34], which can be prepared in cost-effective and large-scale manners. While these mimics may exhibit lower systemic stability compared to agomirs, they still exhibit a certain stability compared to endogenous single-stranded miRNA in insects. Our results showed that they are biologically active in vivo, achieving gene silencing and phenotypic outcomes comparable to agomirs under laboratory conditions. More importantly, they provide a foundation for the high-throughput screening of functional miRNAs at a low cost and future biosynthetic delivery platforms, such as engineered bacteria, fungi, or plants, which can express small RNAs without relying on chemical modifications. These systems may ultimately enable the field-scale, the low-cost application of miRNAs in pest management [35,36].

To explore the miR-71-induced death mechanism, RNA-seq was performed and Transcriptomic analysis revealed that miR-71 had broad-spectrum inhibiting effects on target genes with similar or different function in host response to pathogens. miR-71 downregulates key components of the termite disease defenses, including genes involved in immune response, reactive oxygen species biosynthetic process, glycolysis/gluconeogenesis, citrate cycle (TCA cycle), and olfactory transduction. For example, reactive oxygen species (ROS) production is a frontline defense mechanism in insects, capable of directly killing invading pathogens and signaling downstream immune responses [37,38]. A weakened ROS response may therefore substantially impair the termite’s ability to limit fungal proliferation in the early stages of infection. In addition, miR-71 profoundly impacted host carbohydrate metabolism. The downregulation of genes involved in TCA cycle and glycolysis/gluconeogenesis pathways suggests a systemic reduction in energy availability. This metabolic impairment may compromise the host’s capacity to sustain energetically demanding immune functions, such as melanization, hemocyte proliferation, and antimicrobial peptide synthesis [39,40]. Furthermore, suppression of olfactory transduction genes may diminish environmental sensing, potentially reducing pathogen avoidance behaviors and facilitating fungal transmission within termite colonies [41,42].

Compared to transcriptomic analysis, RT-qPCR results showed that miR-71 mimic exhibited similar silencing effect on several target genes with similar or different function, suggesting that the small silencing RNA can mimic endogenous miRNA, performing the same RNAi function. Among these targets, *collagen alpha-2(IV) chain* is mapped to PI3K-Akt signaling pathway, which plays important roles in processing environmental information [43,44]. *Proclotting enzyme*, *protein croquemort*, and *termicin* are noteworthy due to their known roles in cellular and humoral immune responses [45,46]. The downregulation of these defense-related genes was likely to have negative impact on the behavioral and physiological immunity in termite colonies.

Furthermore, the RNAi-mediated silencing of *hexamerin 1*, *neuroligin-4*, and *probable chitinase* 10 markedly increased the infection mortaltiy of termites, indicating the important resistance to fungal pathogens through physilogical and cuticle resistance in termites. Data showed that miR-71 modulated expressions of the 10 overlapping genes, which determined both transcriptional and translational levels. Among these genes, *hexamerin 1* and *neuroligin-4* have been determined to involve in immune signaling, hemolymph transport, and neural–immune interactions, playing important roles in physilogical resistance to *M. anisopliae* exposure. Probable chitinase 10 is associated with cuticle degradation, whose upregulation may facilitate fungal penetration on termite cuticles. Interestingly, a recent study showed that *M. anisopliae* exposure significantly induced the upregulation of miR-71 expression in termite hosts [15,24]. Here, combined with our findings, we suggested that fungal pathogen might utilize host small-RNA machinery to modulate host mRNA expression, inhibiting physiological resistance and facilitating cuticle penetration.

It is worth noting that although miR-71 upregulated several functional genes, in which some defense-related genes, such as proclotting enzymes, promote host physiological resistance, it still increased the susceptibility of termite hosts to fungal infection. This may be attributed to the fact that the observed phenotype likely arises from the cumulative disruption of multiple defensive systems, consistent with the “barrel theory,” where overall vulnerability is determined by the most compromised pathway. In addition, quantitative proteomics showed that miR-71 downregulated 165 protein expressions, in which only 6 proteins were identified in the transcriptomics findings. This may be because miR-71 cannot completely base pair with most of the genes. In this case, incomplete base-pairing with target genes frequently occurred, resulting in translational inhibition. Therefore, miR-71 is likely to perform posttranscriptional gene silencing mainly through translational inhibition.

## 5. Conclusions

In summary, this study provides proof-of-concept evidence that a T7 RNA polymerase-synthesized, small silencing RNA can functionally replicate the activity of endogenous miR-71 in vivo, enhancing insect susceptibility to entomopathogenic fungi. Specifically, miR-71 mimics significantly promoted *M. anisopliae* virulence against termites by modulating host gene expression across physiological and cuticle resistance. While chemically modified agomirs offer superior stability and delivery characteristics, our findings demonstrate that T7-expressed small RNAs are biologically active similar to insect miRNA and, critically, amenable to low-cost and scalable production for screening functional miRNA and even bioengineering-based pest control.

Although mechanistic insights were supported by multi-omics integration and functional gene-silencing assays, further research is required to confirm direct miRNA–mRNA interactions and to clarify the full regulatory network involved. By combining molecular RNAi strategies with biological control agents, this approach represents a promising, broad-spectrum, and environmentally sustainable alternative to chemical insecticides. It also provides a foundational platform for developing transgenic organisms capable of delivering functional miRNA mimics, thereby offering new directions for precision pest management in agriculture, forestry, and vector control.

## Data Availability

The original contributions presented in this study are included in the article/Supplementary Material. Further inquiries can be directed to the corresponding author.

## Supplementary Materials

The following supporting information can be downloaded at: https://www.mdpi.com/article/10.3390/biom15111517/s1, Table S1: The specific primers of real-time fluorescent quantitative (RT-qPCR). Table S2: Genes identified through RNA-seq in miR-71-treated termites; Table S3: GO analysis of differentially expressed genes in miR-71-treated termites; Table S4: KEGG pathway analysis of differentially expressed genes in miR-71-treated termites; Table S5: Differentially expressed proteins identified by quantitative proteomics in miR-71-treated termites. Table S6: GO term analysis of differentially expressed proteins in miR-71-treated termites; Table S7. KEGG pathway analysis of differentially expressed proteins in miR-71-treated termites.
